# Targeting NMDA receptors in neuropsychiatric disorders by drug screening on human neurons derived from pluripotent stem cells

**DOI:** 10.1038/s41398-022-02010-z

**Published:** 2022-06-09

**Authors:** Wenbo Zhang, P. Joel Ross, James Ellis, Michael W. Salter

**Affiliations:** 1 Program in Neurosciences & Mental Health, The Hospital for Sick Children, Toronto, ON M5G 1X8 Canada; 2 Department of Physiology, University of Toronto, Toronto, ON M5S 1A8 Canada; 3 Biology Department, University of Prince Edward Island, Charlottetown, PE C1A 4P3 Canada; 4 Program in Developmental & Stem Cell Biology, The Hospital for Sick Children, Toronto, ON M5G 1X8 Canada; 5 Department of Molecular Genetics, University of Toronto, Toronto, M5S 1A8 Canada

**Keywords:** Molecular neuroscience, Stem cells

## Abstract

NMDA receptors (NMDARs), a prominent subtype of glutamatergic receptors, are implicated in the pathogenesis and development of neuropsychiatric disorders such as epilepsy, intellectual disability, autism spectrum disorder, and schizophrenia, and are therefore a potential therapeutic target in treating these disorders. Neurons derived from induced pluripotent stem cells (iPSCs) have provided the opportunity to investigate human NMDARs in their native environment. In this review, we describe the expression, function, and regulation of NMDARs in human iPSC-derived neurons and discuss approaches for utilizing human neurons for identifying potential drugs that target NMDARs in the treatment of neuropsychiatric disorders. A challenge in studying NMDARs in human iPSC-derived neurons is a predominance of those receptors containing the GluN2B subunit and low synaptic expression, suggesting a relatively immature phenotype of these neurons and delayed development of functional NMDARs. We outline potential approaches for improving neuronal maturation of human iPSC-derived neurons and accelerating the functional expression of NMDARs. Acceleration of functional expression of NMDARs in human iPSC-derived neurons will improve the modeling of neuropsychiatric disorders and facilitate the discovery and development of novel therapeutics targeting NMDARs for the treatment of these disorders.

## Introduction

Neuropsychiatric disorders, such as epilepsy, intellectual disability (ID), autism spectrum disorder (ASD), and schizophrenia, are complex brain disorders and cause a heavy burden on individuals, family, and society because of the prevalence, life-long course, and few efficacious medications [1–3]. The molecular basis of neuropsychiatric disorders remains incompletely understood, and genetic factors have key roles in the occurrence of these disorders [3, 4]. The imbalance between excitation and inhibition in neural circuits contributes to the pathogenesis and development of various neuropsychiatric disorders [5]. NMDA receptor (NMDAR)-mediated neurotransmission plays a crucial role in excitatory inputs, and therefore these receptors function in maintaining the balance between neural excitation and inhibition in the mammalian central nervous system (CNS) [6]. Dysfunction of NMDARs, which may lead to the imbalance between excitation and inhibition, has been widely reported in neuropsychiatric disorders including epilepsy, ID, ASD, and schizophrenia [7, 8]. NMDARs are therefore increasingly important targets for the discovery and development of drugs. Currently, there are few effective and safe medications for treating neuropsychiatric disorders, and efforts to develop drugs for brain disorders face many challenges [9–11]. The absence of efficacious medications is attributed, in part, to limitations and a shortage of animal models for preclinical studies on the discovery and development of drugs.

Induced pluripotent stem cells (iPSCs) generated by reprogramming human somatic cells have been broadly used since 2008 and afford an efficient model system for disease modeling, toxicity screens, and screening for novel therapeutics because of their genetic relevance to individuals with the disease [12–16]. The neurons derived from human iPSCs have been utilized extensively in neuropsychiatric disorders to probe the molecular pathophysiology of these disorders, and to link molecular findings with clinical features to identify therapeutic targets [2, 12, 17–24]. For example, iPSC studies revealed that ezogabine (an anti-epileptic drug) corrected excitability impairments in a model of amyotrophic lateral sclerosis, leading to subsequent approval for clinical trial [25, 26].

Human iPSC-derived neurons provide a valuable and powerful platform for modeling NMDAR dysfunction associated with neuropsychiatric disorders and translational drug screening because of their relevance to human genetic background and physiology. Disease modeling in iPSCs does not require a priori knowledge of molecular pathways affected in specific neuropsychiatric disorders, and can be used to model effects of variants in specific risk genes or the polygenic risk associated with particular genotypes. Human neurons are advantageous for modeling effects of gene regulatory variants, whose functions may not be conserved between humans and animal models [27, 28]. The expression and importance of human-specific genes in the brain, some of which have been found to be differentially expressed in human iPSC-derived neurons, support the need to use human neurons in investigating neuropsychiatric disorders in combination with mouse models [27]. A recent study reported that a short GluN2A-NMDAR isoform is expressed in brain tissue specifically from human and primate but not rodent [29]. These findings indicate that the human iPSC-derived neuron-based model may be a powerful platform to investigate NMDARs. iPSCs are also powerful for modeling disorders of the brain because they can be differentiated into a wide range of neuronal and non-neuronal subtypes, which can be purified and/or co-cultured in controlled environmental conditions [30]. However, there are challenges to utilize human iPSC-derived neurons for modeling neuropsychiatric disorders, such as relative immaturity of the neurons and delayed development of functional NMDARs [12, 19, 28, 31–33]. It is therefore important to overcome these challenges for utilizing these human neurons for the discovery and development of drugs targeting NMDARs.

## Dysfunction of NMDARs associated with neuropsychiatric disorders

**Fig. 1:**
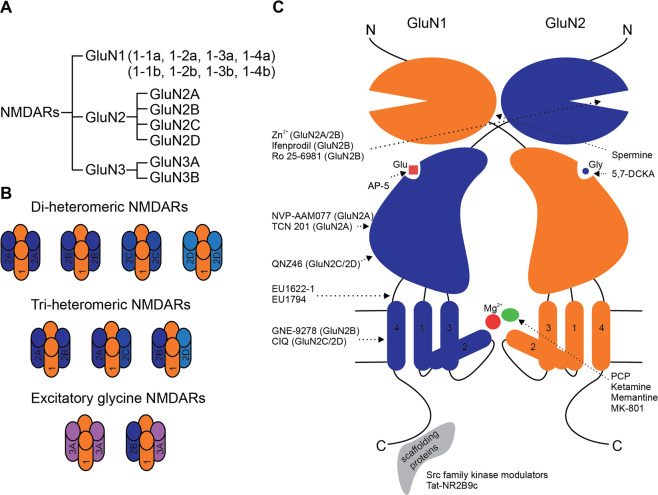
NMDAR subunit composition, structure, and pharmacology. **A** The splice variants of GluN1 subunits and diversity of GluN2 subunits of NMDARs. **B** NMDAR subunit composition. **C** NMDAR structure and pharmacology.

NMDARs, a subtype of ionotropic glutamate receptor, are heterotetrameric cation channels (Fig. 1). The channels contain two obligatory GluN1 subunits with co-agonist glycine binding sites. They also contain two GluN2 subunits with glutamate binding sites, or one GluN2 subunit and one GluN3 subunit [6, 34]. The GluN1 subunits are expressed as eight distinct splice variants encoded by a single *GRIN1* gene, whereas there are four GluN2 subunits (GluN2A-D) encoded by four different *GRIN2* genes [6].

Genetic association studies have identified the biological linkage of hundreds of genetic risk loci with neuropsychiatric disorders, but genetic causal associations in most of these disorders have not been firmly established [4]. However, specific genomic variants associated with dysfunction of NMDARs are implicated in various neuropsychiatric disorders and are widely considered to be one of the molecular bases of the prevalent comorbidities among many of these disorders [7, 8, 35–38]. Dysfunction of NMDARs may result from genomic variations in any of the genes that encode the receptor subunits, but disease-associated variants have been identified primarily in *GRIN1*, *GRIN2A,* and *GRIN2B* [7, 8, 39, 40]. Variants of *GRIN2C-D* and *GRIN3A-B* have also been reported in neuropsychiatric disorders [7, 8, 39]. Reported disease-associated variants of NMDAR subunits include missense, nonsense, frameshift, splice site, and others.

The consequence of these alterations of NMDARs on receptor function and neuronal function is complicated. First, functional analysis shows that these identified genomic variations of NMDARs cause a wide range of changes in NMDAR properties, including potency of agonist/co-agonist, single-channel conductance, receptor kinetics, Ca^2+^ permeability, and pharmacological properties [6, 8]. Second, these variant NMDARs can exhibit hyperfunction (gain of function), hypofunction (loss of function) or no change in function of the receptors in neuropsychiatric disorders. Interestingly, differential types of NMDAR dysfunction may cause similar phenotypes in neuropsychiatric disorders, possibly due to compensatory mechanisms [8]. Some variants of NMDARs occur at the same residue position but cause different types of neuropsychiatric disorders [7, 8]. Finally, genetic variants in other risk genes of neuropsychiatric disorders or other factors can indirectly cause dysfunction of NMDARs as a downstream consequence [41–46]. Here, we outline the dysfunction of NMDARs in neuropsychiatric disorders, and use epilepsy, ID, ASD and schizophrenia as examples to discuss the dysfunction of the receptors.

### Dysfunction of NMDARs associated with neuropsychiatric disorders

#### NMDAR dysfunction in epilepsy

Epilepsy is one of the most common neurological conditions and affects people of all ages [47]. Epilepsy is characterized by unprovoked epileptic seizures, including focal seizures and generalized seizures, due to abnormal electrical activity in neurons, which can cause other health issues in cognition, psychology, and social skills [48]. In addition to clinical features, seizure onset can be confirmed by examining results from electroencephalography and neuroimaging. Alterations in NMDARs, which result in a change in the balance between neural excitation and inhibition, are important cellular events in the pathogenesis of epileptic seizures [7]. Epilepsy-associated variants in NMDAR subunits, including *GRIN1* gene encoding GluN1 subunits and *GRIN2(A-D)* gene encoding GluN2(A-D) subunits, respectively, have been reviewed in detail [8, 49, 50]. In various neuropsychiatric disorders, over 70% of the individuals with *GRIN2A* variants are diagnosed with epilepsy, whereas less than 30% of the individuals with *GRIN2B* variants have epilepsy [7, 8]. The distinct roles of GluN2A and GluN2B in neuropsychiatric disorders may be attributed to the properties of GluN2 subunits in determining the receptor deactivation kinetics, channel gating, Ca^2+^ permeability, and voltage-dependent blockade by extracellular Mg^2+^ [6].

Epilepsy-associated variants in NMDAR subunits have complicated functional consequences, including hyperfunction, hypofunction, and no change in function of the receptors [8, 49]. How such changes in NMDAR function relate to the pathogenesis of epilepsy remains poorly understood.

#### NMDAR dysfunction in ID

ID is a type of neurodevelopmental disorder, a large group of early onset disorders, characterized by deficits in cognitive abilities [51]. In addition to learning disabilities, individuals with ID may have facial features and autistic behavior. Genetic variants are believed to have key roles in the pathogenesis of neurodevelopmental disorders including ID [51], and studies have indicated that variant NMDARs are risk factors for these disorders, implying dysfunction of the receptors in their etiology [7, 8].

*GRIN1* and *GRIN2* variants of NMDARs are implicated in the pathophysiology of ID. However, different from those in epilepsy, *GRIN2B* but not *GRIN2A* variants are mostly associated with ID [8]. In various neuropsychiatric disorders, over 80% of the individuals with *GRIN2B* variants are diagnosed with ID.

#### NMDAR dysfunction in ASD

ASD is a complex neurodevelopmental disorder with cranio-facial characteristics, impairment in social interactions, verbal and non-verbal communication, and restricted, repetitive behavior [51]. Genetic variants of *GRIN1* and *GRIN2* have been implicated in the pathophysiology of ASD, and *GRIN2B* variants are primarily associated with ASD [8]. In various neuropsychiatric disorders, approximately 30% of the individuals with *GRIN2B* variants are diagnosed with ASD.

Alternative splicing of NMDAR GluN1 subunits has been implicated in pathophysiology of ASD [52–54]. The GluN1 subunits have eight distinct isoforms, GluN1-(1a-4a) and GluN1-(1b-4b). The GluN1-b isoforms contain an additional 21 amino-acid sequence, known as the N1 cassette, in an extracellular amino-terminal region of the subunit proteins [6]. The presence or absence of the N1 cassette affects the function and pharmacology of NMDARs [6, 52]. Studies in post-mortem samples of cortex and human iPSC-derived neurons from individuals with ASD have implicated a decrease in expression of *GRIN1-b* isoforms, which contain the N1 cassette [52–54].

In addition, ASD-associated variants of other genes may cause dysfunction of NMDARs through protein-protein interactions, as an important pathophysiological process in the occurrence of the disorders. These genes include *NLGN*, which encodes the protein neuroligins [55–58], and *SHANK* genes, which encode postsynaptic scaffolding proteins of the SHANK family [59–63]. Autism-associated disruption of the long noncoding RNA, *PTCHD1-AS*, leads to dysfunction of NMDARs [64].

#### NMDAR dysfunction in schizophrenia

Schizophrenia is a major, chronic psychiatric disorder and is characterized by hallucinations, disorganized thoughts, cognitive dysfunction, and attention and social deficits [65]. Schizophrenia is a highly heritable psychiatric disorder and has a genetic origin, with a high prevalence of approximately 1% of the population worldwide [65]. Genome-wide association studies have provided insights into numerous risk genes for schizophrenia implicating the protein products in the pathogenesis of this severe psychiatric disorder [66–70]. Variants in NMDAR subunits, which cause hypofunction of NMDARs, have been implicated in the pathophysiology of schizophrenia [7, 8, 66, 70–75].

Before the surge of genetic evidence supporting the association of NMDAR malfunction with schizophrenia, hypofunction of NMDARs in the pathogenesis of schizophrenia was originally identified from observations on the psychotomimetic effects of non-competitive NMDAR antagonists. These antagonists including phencyclidine- and ketamine-induced symptoms similar to those of human subjects with schizophrenia [76].

In addition, hypofunction of NMDARs has been reported in schizophrenia due to alterations in signaling pathways through NMDAR-interacting proteins such as neuregulin-ErbB4 receptors [42, 43, 77], disrupted-in-schizophrenia 1 (DISC1) [44, 78], calcineurin [79], and α7 nicotinic acetylcholine receptors [45, 80].

### Screening of drugs targeting NMDARs in neuropsychiatric disorders

Complicated consequences on NMDAR function including hyperfunction, hypofunction, and no change in function, which are caused by genetic variants of these receptors in neuropsychiatric disorders such as epilepsy, ID, ASD, and schizophrenia, lead to the need for specific drugs targeting these receptors for treating these disorders. Thus, a mechanism-based approach, screening of specific drugs targeting NMDARs, could be advantageous for novel therapeutics in neuropsychiatric disorders, and human iPSC-derived neurons can provide an unprecedented system for modeling these disorders and the screening.

### Challenges of human iPSC-derived neurons in modeling neuropsychiatric disorders

Human iPSC-derived neuron-based disease models are a promising strategy for identifying novel therapeutics because they are genetically matched to individuals with neuropsychiatric disorders [81, 82], and therefore provide a unique opportunity as a translational screening platform for the discovery and development of drugs targeting NMDARs. In addition, human iPSC-derived neuron-based model is a promising strategy for neurotoxicity screening. Human iPSC-derived neurons have biological and physiological relevance to human primary neurons and thus provide a valuable and powerful platform for neuronal toxicity studies by improving the accuracy of screening for therapeutic compounds as well as decreasing the cost and time-consuming in pre-clinical trials [83]. Despite advantages in disease modeling, studies also revealed the presence of challenges to utilize human iPSC-derived neurons for modeling neuropsychiatric disorders, which include a relatively immature feature of the neurons, variation between iPSC lines, neuronal density-induced variability, and very slow differentiation into functional neurons [12, 19, 28, 31–33].

Over the past decade, approaches for improving the maturation of these neurons have been explored. Rapid single-step neurogenin-2 (NGN2) induction accelerates the maturation of neurons directly converted from iPSCs without a stage of neural progenitor cells (NPCs), which mature in 3–4 weeks [84]. In addition, compared with conventional two-dimensional cell culture systems, three-dimensional cell culture systems—brain organoids—may more closely mimic the environment of neurodevelopment in the mammalian CNS and thus improve the maturation of iPSC-derived neurons [85–87]. However, organoids show challenges and disadvantages such as experimental cost, technical issues for imaging and electrophysiology, and variability between batches [85, 88].

Maturation of human iPSC-derived neurons can be promoted through modifying culture environments. Co-culture with astrocytes accelerates functional maturation and synapse formation of human iPSC-neurons [89–94], and thus improves the efficiency for modeling neuropsychiatric disorders [18, 64, 95–97]. Functional maturation of human iPSC-derived neurons is also promoted by using culture media such as BrainPhys [98, 99]. In addition, nanoscale biophysical stimulation could promote the maturation of human iPSC-derived neurons through substrate nanotopography [100, 101].

Variability between cultures in neuronal density may affect neuronal and network characteristics. This may be at least partially controlled by co-culture of iPSC-derived cells from unaffected controls together with those from individuals with neuropsychiatric disorders [95, 97]. Such co-culture also helps to overcome the challenge of the effects of varying genetic background between cultures and, in addition, provides a chance to examine whether alterations are cell autonomous [95, 97, 102].

Another challenge associated with disease modeling in iPSCs is limited statistical sensitivity arising from variability and limited sample sizes [24, 28, 103]. Standard guidelines dictate that iPSC studies of brain disorders should include ≥2 affected individuals and ≥2 unaffected controls, with 2–3 clonal iPSC lines per person [24]. Numbers of iPSC lines are often limited by cost, time, or availability (particularly for rare disorders). When many donors are available, statistical power may be improved by increasing the number of individuals in the study at the expense of analyzing fewer iPSC clones per individual [103]. To overcome variability between donors, genome editing can be used to introduce targeted mutations in specific neuropsychiatric risk genes, and subsequent analyses of isogenic lines may also improve statistical sensitivity [24, 28, 103]. Other approaches to overcome variability include limiting variability between study participants by focusing on individuals with similar genetic variants (e.g., loss of function variants in *GRIN2B*), or individuals with defined phenotypic classifications (e.g., NMDAR hypofunction). Some experimental approaches like transcriptomics are particularly sensitive to heterogeneity, so computational tools have been developed to improve signal-to-noise ratio [104]. RNA deconvolution may be a potential method to eliminate transcriptomic variability in neural differentiation and maturation [91].

### Human NMDARs in situ in iPSC-derived neurons

#### Expression level of human NMDARs in situ

**Fig. 2:**
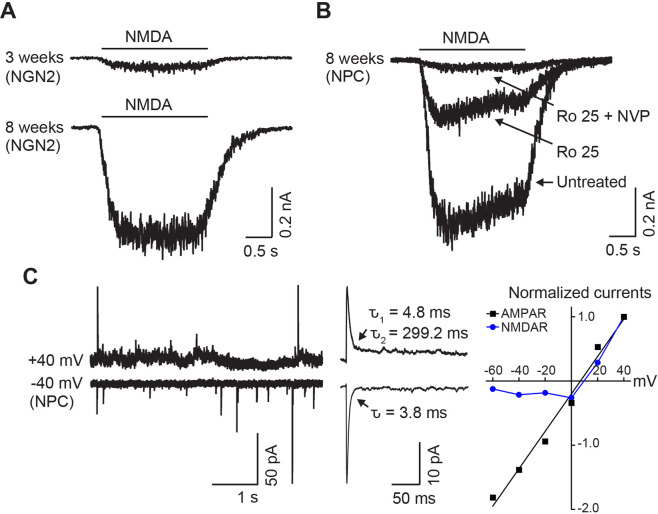
NMDAR currents in human iPSC-derived neurons. **A** Traces displaying NMDA-evoked currents in 3- and 8-week-old human NGN2 iPSC-derived neurons, which were differentiated with a rapid single-step NGN2 induction. **B** A representative trace shows NMDA-evoked currents in a human iPSC-derived neuron, which was differentiated through an NPC stage in 8-week-old culture, and the effects of Ro 25-6981 (1 µM), a selective antagonist of GluN2B-containing NMDARs and NVP-AAM077 (0.4 µM), an antagonist of GluN2A-containing NMDARs. **C** Left, Traces showing mEPSCs recorded at the membrane potentials of −40 mV (bottom) and +40 mV (upper) in presence of extracellular Mg^2+^ at 2 mM in a human iPSC-derived neuron, which was differentiated through an NPC stage in about 4-month-old culture. Middle, Averaged mEPSCs at −40 mV and +40 mV in this cell. Right, A plot depicting the current-voltage relationship of AMPA-mEPSCs and NMDAR-mEPSCs in this cell.

Human iPSC-derived neurons progressively express functional NMDARs over time in culture (Fig. 2). The amplitudes of NMDA-evoked currents are substantial in neurons with about 8 weeks of differentiation in culture after the NPC stage, but synaptic NMDAR currents may be absent at this time and require even longer-term culture [32, 64, 97, 105, 106]. In human NGN2 iPSC-derived neurons, in which the NPC stage is skipped, NMDAR currents are tiny or undetected, although AMPA receptors, another major type of ionotropic glutamate receptor, are robustly expressed (Table 1) [84, 107, 108]. This contrasts to normal development of glutamatergic synapses, during which expression of NMDARs is prior to that of AMPA receptors and AMPA receptors are inserted into NMDAR-dominant excitatory synapses called “silent synapses” [109–111]. Thus, skipping the NPC stage in the induction of human NGN2 iPSC-derived neurons appears to cause a loss of a developmental window, thereby leading to decreased expression of NMDARs. In addition, human NGN2 iPSC-derived neurons express very large voltage-gated Na^+^ currents and voltage-gated K^+^ currents [84], reinforcing the limited expression of NMDARs in these neurons (Table 1). Extrasynaptic NMDAR currents in human NGN2 iPSC-derived neurons may be able to be enhanced through long-term culture, but even then, it is difficult to detect synaptic NMDAR currents [84, 107, 108].

**Table 1:** Comparison of NMDAR currents with AMPAR currents and other ion channel-mediated currents in the previously reported studies in human iPSC-derived neurons and in neurons in the human brain biopsy tissue.

Authors	Year	Protocol	Weeks in culture	Neuron type	NMDAR		AMPAR (pA)	Na^+^ channel (pA)	K^+^ channel (pA)
					mRNA	Current (pA)			
Ross et al. [64]^a^	2020	NPC	8–16	Mixed		−1000	−10	−1500	1400
Zhang et al. [32]	2016	NPC	>7	Mixed	+	−600	−400	−1300	1900
Shcheglovitov et al. [97]	2013	NPC	5–6	Mixed	+	−37	−52		
						−80	−70		
Zhang et al. [84]^b^	2013	NGN2	>2	excitatory	–	0	−400	−2200	2500
Lam et al. [108]^c^	2017	NGN2	2–6			−0.5	−7	−55	107
Nehme et al. [107]^d^	2018	NGN2	3–7	excitatory	+	0	−50	−1600	1400
						−75			
Pegasiou et al. [160]	2020	Human brain				−100	−95		

Data displayed in this table are approximate values reported in the previously reported studies. If there are two rows from the same study, synaptic currents are displayed in the upper one and NMDA-evoked currents are displayed in the bottom one. Unless otherwise indicated, NMDAR currents and AMPAR currents were evoked by applying NMDA and AMPA, respectively, in human iPSC-derived neurons, which were differentiated through an NPC stage (NPC) or differentiated with a rapid single-step NGN2 induction (NGN2).

^a^AMPAR-mEPSCs were recorded.

^b^Synaptic AMPAR- and NMDAR-currents in 3-week-old neurons.

^c^Current density (pA/pF) in 4-week-old culture.

^d^NMDA-evoked currents in 3-week-old *CAMK2A*+ neurons.

#### Subunit composition of human NMDARs in situ

mRNA expression of NMDAR subunits, including *GRIN1*, *GRIN2(A-D)*, and *GRIN3(A-B)*, has been probed in human iPSC-derived neurons [95, 112, 113]. Electrophysiological studies uncovered that GluN2A subunits mediate only 10–30% of NMDA-evoked currents, whereas GluN2B subunits mediate over 70% of these currents in both human iPSC-derived neurons differentiated through an NPC stage and in NGN2 neurons [32, 112, 114], suggesting that GluN2B subunits dominate NMDAR responses in these neurons. Furthermore, the decay time of NMDAR-mediated miniature excitatory postsynaptic current (mEPSCs) is beyond 250 ms [32], confirming that GluN2B subunits, which have much slower decay time kinetics than GluN2A subunits [6], are the dominant subtype of NMDAR in human iPSC-derived neurons. The subunit diversity of NMDARs determines the receptor properties and function in the physiological and pathological processes in the mammalian CNS [6]. The GluN2 subunits have differential expression and distribution in specific brain regions, neuronal types, and locations. Furthermore, NMDAR subunits, specifically GluN2A and GluN2B, display unique spatiotemporal expression patterns [6]. GluN2A and GluN2B dominate in the mammalian higher brain structures such as the cortex, and it is well known that there is a developmental switch for NMDAR GluN2 subunits during postnatal development [6], which leads to a change of NMDAR subunit composition from GluN2B to GluN2A subunit-dominant receptors in most brain regions including the cortex, particularly at synapses. This switch is evolutionarily conserved and related to synaptic maturation and learning formation [6]. Thus, the finding of GluN2B subunit-dominant NMDARs suggests that there is the delay of the developmental trajectory of NMDARs in human iPSC-derived neurons.

GluN2C subunits have not been detected at the protein level in human iPSC-derived neurons, whereas these neurons express GluN2D subunit proteins at a very low level, with no function [112]. Currently, there are no studies investigating NMDAR GluN3 subunit proteins or function in human iPSC-derived neurons.

#### Pharmacological properties of human NMDARs in situ

Human NMDARs in situ in iPSC-derived neurons have pharmacological properties similar to those of neurons in the mammalian CNS, such as voltage-dependent block by extracellular Mg^2+^, sensitivity to Zn^2+^, and blockade by TCN 201 and NVP-AAM077 of GluN2A antagonists and Ro 25-6981 and ifenprodil of GluN2B antagonists [32, 105, 112]. In addition, NMDAR currents are potentiated in human iPSC-derived neurons by GNE-9278, a selective, positive allosteric modulator of GluN2B subunits [112]. Moreover, NMDAR currents are upregulated by Fyn kinase, a member of the Src family of non-receptor tyrosine kinases, in human iPSC-derived neurons, as in the neurons from the rodent CNS [32, 115].

Thus, human iPSC-derived neuron-based models can be utilized for investigating NMDARs, and studies have uncovered the association of the receptor deficiency with neuropsychiatric disorders with this model [52, 64, 95, 97, 113, 116–119]. However, the delay of the developmental trajectory of NMDARs, such as low expression of GluN2A and limited synaptic localization of these receptors, may hamper the utilization in disease modeling and the discovery and development of drugs targeting NMDRs, especially for the receptor subtype-selective targeting.

## Promoting neuronal maturation and accelerating functional expression of NMDARs in human iPSC-derived neurons

### Optimizing differentiation to promote maturation of human iPSC-derived neurons

We have learned a lot from studying animal models, in particular, that small molecules significantly accelerate neuronal differentiation in rodent primary neural cultures [120, 121]. In addition, small molecules promote the maturation of neurons derived from rodent embryonic stem (ES) cells, as well as from human ES cells [122–125]. These studies provide a strong basis for utilizing small molecules to promote the neuronal maturation of human iPSC-derived neurons [126–130].

Efforts using high-throughput screen for compounds with human iPSC-derived neurons have identified over 100 small molecules, including 37 approved drugs, which modulate neurite growth [131]. Combinatorial application of small molecules, including DAPT, PD0315901, SU5402, and CHIR99021, accelerated the induction of early-born, human iPSC-derived neurons with mature features including electrophysiological properties, and the resulting neurons were able to form long-distance projections in the postnatal mouse cortex after they were transplanted at 8 days of differentiation [128]. Azidothymidine, an inhibitor of telomerase, improves differentiation and maturation of human iPSC-derived neurons [132]. Modulating gene expression is an alternative approach for improving the maturation of human iPSC-derived neurons. Expression of the transcription factor achaete-scute homolog 1 improves the maturation of human iPSC-derived neurons through increasing neurite length and numbers of branch points [133].

### Facilitating functional expression of NMDARs in human iPSC-derived neurons

Thrombospondin-4 (THBS4) and SPARC-like protein 1 (SPARCL1), specific factors enriched in serum from young mice, improve synaptic connectivity of neurons differentiated from human ES cells [134]. In particular, SPARCL1 but not THBS4 promotes functional expression of NMDARs and such improvement occurs specifically at excitatory but not inhibitory synapses [134, 135].

The developmental switch of NMDAR subunits from primarily GluN2B to GluN2A shapes synaptic plasticity and learning and memory formation [6]. The repressor element 1 silencing transcription factor (REST) can trigger the developmental switch of NMDAR GluN2 subunits in the hippocampus of neonatal rats [136], and cellular prion proteins are required for the REST-regulated developmental switch [137]. Furthermore, miR-19a and miR-539, two microRNAs which modulate gene expression post-transcriptionally, collaborate with REST and thus regulate the expression of NMDAR GluN2A and GluN2B subunits in rat primary neurons [138]. Homeodomain interacting protein kinase 2 (HIPK2) negatively regulates the expression of NMDAR subunit GluN2A and GluN2C and knockout of *Hipk2* in the mouse brain increases the expression of GluN2A and thus the GluN2A/GluN2B ratio through altering JNK (c-Jun N-terminal kinase)-c-Jun signaling pathway [139]. In addition, the activity of histone deacetylase has a role in regulating the expression of GluN2B in rat cortical neurons and thus may have an effect on the developmental switch of NMDAR subunits and synaptic maturation [140].

Metabotropic glutamate receptor type 5 (mGluR5) has a role in the developmental switch of NMDAR subunits via phospholipase C signaling pathway, and knockout of mGluR5 abolishes the developmental switch in mouse primary neurons [141]. In addition, casein kinase 2, a serine/threonine protein kinase, causes GluN2B endocytosis from synapses through the phosphorylation in the PDZ binding domain of the subunits (S1480) and thus allows synaptic incorporation of GluN2A in rat primary neuronal cultures [142]. Such a process of phosphorylation is enhanced by activation of Ca^2+^/calmodulin-dependent protein kinase II (CaMKII), which functionally couples with casein kinase 2 and GluN2B [143].

The approaches above in animal models to drive the developmental switch of NMDAR GluN2 subunits are worth examining whether they can induce the switch and synapse maturation in human iPSC-derived neurons. In addition, the developmental switch is activity and sensory experience-dependent [144], and therefore increasing neuronal activity by depolarization or optogenetic stimulation may have the potential to drive the subunit switch in human iPSC-derived neurons. In mouse interneurons, gene expression changes during the first weeks of postnatal maturation are largely regulated by activity-dependent transcription factors like Fos [145]. The activity-dependent enhancers that regulate maturation-associated genes are activated between 1 and 3 weeks after birth and then remain active in aged mice, suggesting these activity-driven expression changes are permanent. Sensory experience is a major source of neuronal activity during the first weeks of postnatal development. Some of these sensory stimuli naturally fluctuate in daily cycles, and circadian entrainment has been shown to promote functional maturation of human embryonic stem cell-derived beta cells [146]. Therefore, in addition to testing whether neuronal activity can promote maturation-associated changes in NMDAR subunits, it may be interesting to test whether circadian entrainment by timed activity can promote the NMDAR subunit switch.

## Human iPSC-derived neurons as a translational screening platform for the discovery of drugs targeting nmdars

### Feasibility of using human iPSC-derived neurons for screening of drugs targeting NMDARs

Recent investigations have indicated the feasibility of using human iPSC-derived neurons for the discovery and development of drugs targeting NMDARs. High content imaging was utilized to measure the effect of compounds on neurite growth of human iPSC-derived neurons and approximately 100 out of 4421 bioactive small molecules were identified, which control neurite growth [131]. Such high content imaging may be a suitable approach to high-throughput screening of drugs modulating the expression of NMDARs in human iPSC-derived neurons.

The permeability to Ca^2+^, as well as to monovalent cations like Na^+^ and K^+^, allows Ca^2+^ imaging to be a reliable, but indirect method to measure NMDAR function, and high content imaging-based analysis of intracellular Ca^2+^ dynamics can evaluate NMDAR-mediated Ca^2+^ signaling for identifying drugs targeting the receptors. Another approach to investigate NMDAR function is the use of microelectrode arrays (MEA), which record neuronal spiking activity extracellularly. Monitoring the level of intracellular Ca^2+^ and MEA provide powerful, high-throughput approaches to examine neural circuit activity. Ca^2+^ imaging [147] and MEA [113] have been successfully utilized in human iPSC-derived neurons for readout of NMDAR function through evaluating Ca^2+^ signaling and contribution of synaptic transmission to network activity. However, Ca^2+^ imaging and MEA as indirect approaches, have limitations on examining NMDAR function, particularly on pharmacological studies on NMDARs. To overcome the limitations, promising compounds identified using these approaches can then be tested using whole-cell patch-clamp electrophysiology for directly exploring the molecular mechanisms underlying modulation of NMDAR function.

**Fig. 3:**
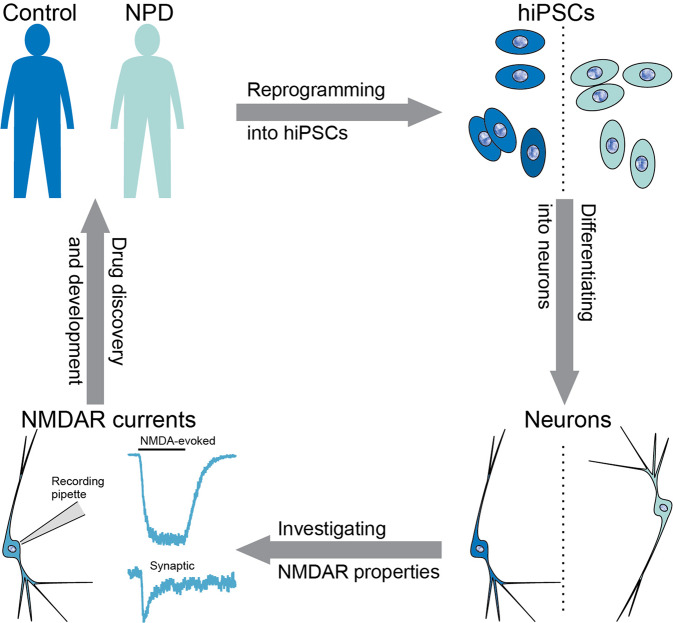
A schematic depicting utilization of human iPSC-derived neurons for modeling dysfunction of NMDARs associated with neuropsychiatric disorders. Human neurons differentiated from iPSCs from individuals with neuropsychiatric disorders (NPD), with or without an NPC stage, are used for translational screening platform and for the discovery and development of drugs targeting NMDARs. NMDA-evoked currents and synaptic NMDAR currents are investigated in human iPSC-derived neurons from unaffected controls and individuals with neuropsychiatric disorders.

Based on these observations, a few powerful, high-throughput approaches are feasible for unbiased phenotyping and screening of drugs targeting NMDARs in neuropsychiatric disorders (Fig. 3).

### Targeting NMDARs for discovery of drugs using human iPSC-derived neurons

Human NMDARs in situ in iPSC-derived neurons provide an opportunity for their use in drug screening in treating neuropsychiatric disorders. The strategies of drug development on NMDARs are identifying new compounds or repurposed drugs directly targeting NMDARs, or indirectly affecting NMDAR function or signaling pathways downstream of the receptors.

#### Targeting NMDARs directly

Up to now, there has been limited clinical success in drugs directly targeting NMDARs and a few modulators of NMDARs such as memantine and ketamine have been approved by FDA for clinical use despite considerable numbers of filed patents on compounds or drugs related to NMDARs [148]. The primary challenges in the development of drugs directly targeting NMDARs are lack of personalized therapeutics, subtype-selective targeting, and adverse or psychiatric side effects.

As both hyperfunction and hypofunction of NMDARs are implicated in the pathophysiology of epilepsy and ASD [8, 41], a more mechanism-based approach will be advantageous for personalized therapeutics. With the loss of function of NMDAR, the strategy is to enhance NMDAR activity [8]. A recently identified positive allosteric modulator, thienopyrimidinone EU1622-1 restores NMDAR function but does not cause excessive Ca^2+^ influx and excitotoxicity through increasing agonist potency and channel open probability but diminishing single-channel conductance and calcium permeability [149]. With gain of function of NMDARs, the strategy is to use NMDAR antagonists such as memantine, dextromethorphan, and ketamine to reduce NMDAR activity for avoiding the receptor-mediated excitotoxic brain injury [150].

Subtype-selective targeting of NMDARs has been important to the discovery and development of drugs. Non-subunit selective NMDAR compounds have a broad-spectrum blockade but cause a number of adverse or psychiatric side effects in the mammalian CNS [148, 150]. Thus, NMDAR drug development takes advantage of unique pharmacological properties of the receptor subunits for subtype-selective targeting. In addition, developing NMDAR modulators with low-affinity or partial effects might be a promising strategy because this reduces the psychiatric side effects [150].

Another challenge is to identify drugs discriminatively targeting synaptic and extrasynaptic NMDARs because they have different roles in brain function and in the pathogenesis of neuropsychiatric disorders [6]. An allosteric modulator of NMDARs, EU1794, has bidirectional effects on the receptors, dependent on the concentrations of both glutamate and glycine [151]. EU1794 exhibits a potentiation on NMDARs at a low concentration of glutamate but has an inhibitory effect at a high glutamate concentration. Thus, EU1794 may have the potential to exhibit differential effects on NMDARs between synaptic and non-synaptic sites because of the difference in the concentration levels of glutamate, at a high concentration synaptically and at a low concentration extrasynaptically [151].

#### Targeting NMDAR-associated signaling complexes

NMDARs form a large multiprotein complex through protein-protein interactions and therefore modulating NMDAR function through the interventions of the interactions is a strategy for the discovery and development of drugs that indirectly affect NMDAR function or signaling pathways downstream of the receptors [6, 152, 153]. Here, some of the NMDAR-associated signaling pathways are highlighted as targets for drug screening.

Neuregulin-ErbB4 receptors signaling pathways are implicated in the pathophysiology of schizophrenia [42, 43, 77, 154]. Neuregulin-1 (NRG1), encoded by the schizophrenia candidate gene *NRG1*, binds to its receptors, ErbB4, and activates the receptors. ErbB4 activation abolishes the increase of NMDAR activity induced by members of the Src family kinases and suppresses long-term potentiation [43, 155, 156]. Members of the Src family kinases regulate human NMDARs in situ in iPSC-derived neurons [32], and therefore Src family kinases as NMDAR-associated signaling complexes could be a target for promising therapeutics in treating neuropsychiatric disorders.

Other proteins, including CaMKII and the postsynaptic density protein PSD-95, interact with NMDARs and may be promising for the translational screening of drugs targeting NMDARs in the treatment of neuropsychiatric disorders. CaMKII, a serine/threonine protein kinase, plays a key role in NMDAR-dependent long-term potentiation and long-term depression [6]. In addition, CaMKII has been implicated in the pathogenesis of various neuropsychiatric disorders [157]. PSD-95, a scaffolding protein, directly interacts with NMDARs to modulate the receptor activity, and could therefore be a potential molecular target in the development and discovery of drugs [6]. Tat-NR2B9c, an interfering peptide, dissociates NMDARs from PSD-95 [158] and improves outcomes after ischemic stroke [159], providing proof of concept that targeting interactions in the NMDAR complex can have therapeutic benefits in humans.

Associated signaling complexes of NMDARs may therefore be promising therapeutic targets for the development and discovery of drugs. Such therapeutics, compared with direct agonists or antagonists of NMDARs, may have mild effects on the receptors per se, thus reducing adverse or psychiatric side effects.

## Conclusions

NMDARs have been implicated in pathophysiology of neuropsychiatric disorders, and therefore modeling NMDAR dysfunction with human iPSC-derived neurons may lead to an unprecedented opportunity to identify novel therapeutic drugs. Efforts to obtain mature human iPSC-derived neurons will make the neurons more broadly applicable for disease modeling. In particular, driving functional expression of NMDARs in human iPSC-derived neurons will contribute to investigations on NMDARs and screening for drugs targeting these receptors, and foster the discovery and development of drugs for treating neuropsychiatric disorders.
